# Quantifying Uncertainties in N_2_O Emission Due to N Fertilizer Application in Cultivated Areas

**DOI:** 10.1371/journal.pone.0050950

**Published:** 2012-11-30

**Authors:** Aurore Philibert, Chantal Loyce, David Makowski

**Affiliations:** 1 INRA, UMR 211 Agronomie, Thiverval Grignon, France; 2 AgroParisTech, UMR 211 Agronomie, Thiverval Grignon, France; University of Illinois, United States of America

## Abstract

Nitrous oxide (N_2_O) is a greenhouse gas with a global warming potential approximately 298 times greater than that of CO_2_. In 2006, the Intergovernmental Panel on Climate Change (IPCC) estimated N_2_O emission due to synthetic and organic nitrogen (N) fertilization at 1% of applied N. We investigated the uncertainty on this estimated value, by fitting 13 different models to a published dataset including 985 N_2_O measurements. These models were characterized by (i) the presence or absence of the explanatory variable “applied N”, (ii) the function relating N_2_O emission to applied N (exponential or linear function), (iii) fixed or random background (i.e. in the absence of N application) N_2_O emission and (iv) fixed or random applied N effect. We calculated ranges of uncertainty on N_2_O emissions from a subset of these models, and compared them with the uncertainty ranges currently used in the IPCC-Tier 1 method. The exponential models outperformed the linear models, and models including one or two random effects outperformed those including fixed effects only. The use of an exponential function rather than a linear function has an important practical consequence: the emission factor is not constant and increases as a function of applied N. Emission factors estimated using the exponential function were lower than 1% when the amount of N applied was below 160 kg N ha^−1^. Our uncertainty analysis shows that the uncertainty range currently used by the IPCC-Tier 1 method could be reduced.

## Introduction

Nitrous oxide (N_2_O) is a greenhouse gas (GHG) with a global warming potential approximately 298 times greater than that of CO_2_ [1]. N_2_O emissions increased by almost 17% from 1990 to 2005 [2]. The nitrogen (N) cycle is complex and N_2_O emissions are determined by many factors [3]. Natural and anthropogenic N_2_O is emitted as a result of nitrification (oxidation of ammonia) and denitrification (nitrate reduction), and these processes are influenced by applications of mineral N fertilizer and manure to agricultural soils [4], [5]. N applications are recognized as the major source of anthropogenic nitrous oxide emission [6], [7]. N_2_O emissions are also influenced by other management practices (e.g., tillage [8]), soil and climate characteristics (e.g. soil water content) [9], [10], [11], [12], [13].

For countries unable to provide local statistics, N_2_O emission can be estimated by the IPCC-Tier 1 method. In this approach, direct N_2_O emission from N inputs is calculated as Y_N inputs_ = (F_SN_+F_ON_+F_CR_+F_SOM_)*EF_1_+ (F_SN_+F_ON_+F_CR_+F_SOM_)_FR_*EF_1FR,_ where F_SN_ is the annual amount of synthetic N fertilizer applied to soils, F_ON_ is the annual amount of organic N applied to soils, F_CR_ is the annual amount of N in crop residues, F_SOM_ is the annual amount of N in mineral soils, EF_1_ is the emission factor for N_2_O emissions from N inputs and FR indicates that the value concerned is for flooded rice [14]. For all crops other than flooded rice, the relationship between N_2_O emission from N fertilizer and the dose of N applied can be expressed as Y = EF*X, where Y represents N_2_O emissions due solely to N fertilization, X is the amount of synthetic and organic N applied and EF (emission factor) is the amount of N_2_O emitted per unit of applied N. In the United Nations Framework Convention on Climate Change [15], 56% of developed countries reported using the Tier 1 method of the IPCC to estimate N_2_O emission from agricultural soils in 2006, and half the published N_2_O emission inventories are based on this approach [16].

The EF value of 1.25%, set in 1999 [17], was calculated from the following linear regression: Y = 0.0125*X, where Y is the emission rate (in kg N_2_O-N ha^−1^ yr^−1^) and X is the fertilizer application rate (in kg N ha^−1^ yr^−1^), based on 20 experiments [18]. A background emission of 1 kg N_2_O-N ha^−1^ yr^−1^ (i.e., emission for X = 0) was obtained in five experiments. The new value of EF used by the IPCC after 2006 (1%; [14]) was estimated from a larger dataset, including N_2_O emission measurements from studies on both crops and grassland [10].

Several recent studies have improved the estimation of N_2_O emission further. Process-based models, such as the DNDC model [12] have been used to calculate the N_2_O emission factor as a function of the organic carbon content of the soil, fertilizer type and weather conditions, and the DAYCENT model [19] has been used to calculate N_2_O emissions as a function of soil class, daily weather, historical vegetation cover and land management practices, such as the type of crop grown, fertilizer additions and cultivation events. As these models describe the nitrogen cycle in detail, they may require long computation times and many input variables and are therefore difficult to implement [20]. Various statistical models have also recently been proposed for the estimation of N_2_O emission from global datasets. For example, linear regression models have been used [20], [21], and a nonlinear model based on an exponential function was proposed in another study [13].

The IPCC-Tier 1 method used for the estimation of N_2_O emissions due to N fertilization includes three main sources of uncertainty on N_2_O emission: (i) the uncertainty concerning the equation relating N_2_O emission to applied N, (ii) the uncertainty concerning the equation parameters and (iii) the uncertainty about the amount of applied N (X).

In the IPCC-Tier 1 method, N_2_O emission is assumed to be linearly related to applied N, but this assumption has been challenged; some authors [22], [23] suggest that N_2_O emission may instead increase exponentially as a function of applied N, and an exponential relationship between Y and X was also considered in the N_2_O mitigation protocol proposed by Millar et al. [24]. A nonlinear relationship between Y and X was also considered by Stehfest and Bouwman [10]. There is currently no consensus concerning the most appropriate function for describing the relationship between N_2_O emission and applied N at the global scale.

Uncertainty about the true value of the model parameter EF is another source of concern, for two reasons. First, N_2_O emission measurements are known to be highly variable, both within a given site-year and between site-years. For a given site-year, N_2_O emission varies principally due to climatic conditions, such as variations in the timing and intensity of rainfall, which modify microbial activity and the rates of gaseous emission [25]. For example, N_2_O emissions must be measured after a period of rain to detect peaks in emission. Many factors may be responsible for variability between site-years, including differences in management practices (e.g. type of N fertilizer), soil characteristics and weather conditions between sites and years [20], by modifying chemical exchanges in agricultural soils. Duration of the experiment [10] and method used to measure emissions [11] may also affect N_2_O emission measurements. Second, the emission factor can be estimated by several different statistical methods, some based on fixed-parameter models (i.e., classical regression) and others based on mixed-effect models or Bayesian methods. The sensitivity of EF to the statistical method used for its estimation has never been evaluated.

Finally, the amounts of N applied can be estimated from regional and national statistics and from interviews with farmers [10], [26], [27], but the actual amounts of N applied are not perfectly known and vary from year to year.

In this study, we focused on the first two of these sources of uncertainty: the equation of the model and the values of the model parameters. We fitted 13 different models to the dataset of Stehfest and Bouwman [10], and calculated uncertainty ranges on average N_2_O emissions from a subset of these models, comparing our ranges with those currently used by the IPCC.

## Materials and Methods

### Database

The dataset is a global compilation of nitrous oxide (N_2_O) and nitric oxide (NO) emissions extracted from peer-reviewed publications appearing between 1979 and 2004, established by Stehfest and Bouwman [10]. Readers should refer to the original paper by Stehfest and Bouwman for a more complete presentation of the data.

The dataset (available from http://www.pbl.nl/en/publications/2006/N2OAndNOEmissionFrom AgriculturalFieldsAndSoilsUnderNaturalVegetation) includes 1891 measurements of N_2_O and NO emissions in natural and agricultural fields from 387 publications. As we focused on calculation of the emission factor associated with fertilizer applications in agricultural fields (EF), we excluded the following experiments from the initial dataset: (i) 418 experiments carried out in natural areas, (ii) 360 experiments including measurements of NO emission only, (iii) 57 experiments on organic soils (not concerned by EF), (iv) 25 experiments including the use of chemicals or additives considered to inhibit nitrification (also excluded by Stehfest and Bouwman [10]), (v) 8 experiments in grazing systems (also excluded by Stehfest and Bouwman [10]), (vi) 38 experiments in which the amounts of applied N exceeded 500 kg N ha^−1^ yr^−1^ (given that the maximum amounts of N applied to agricultural fields has been estimated at 400 kg N ha^−1^ [20], [27], [28]).

**Figure 1:**
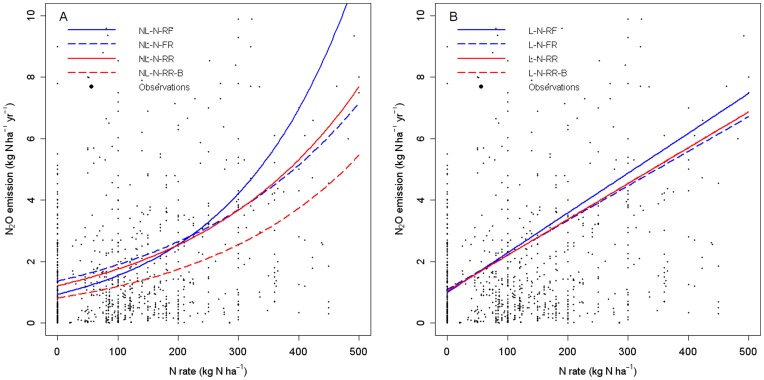
Fitted response curves obtained with the four selected nonlinear models (A) and the four selected linear models (B). Black points correspond to N_2_O data (96.04% of available observations are displayed; the other data are too extreme for graphical presentation).

**Figure 2:**
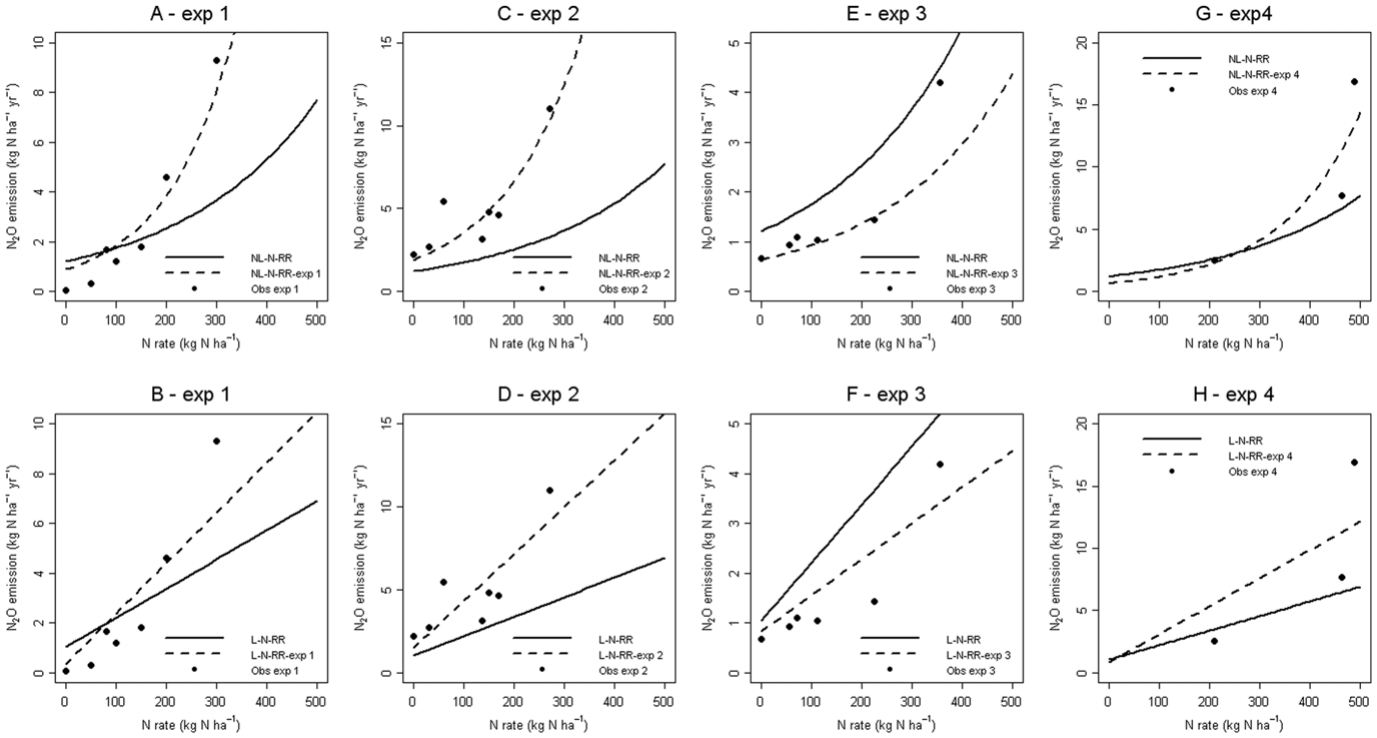
Fitted response curves for four experiments (exp 1: (A–B), exp 2: (C–D), exp 3: (E–F) and exp 4: (G–H)). For each published-experiment, mean response (solid black line) and experiment-specific response (dotted black line) were calculated with model NL-N-RR (A, C, E, G) and model L-N-RR (B, D, F, H). Black points represent N_2_O data averaged over replicates.

We finally worked with a dataset including 985 measurements of N_2_O emission in agricultural fields extracted from 203 publications, corresponding to a set of experiments encompassing various soil and climatic characteristics and types of fertilization (Figs. 1 and 2).

The distribution of N_2_O measurements and amounts of applied N are presented in Table 1 for the entire dataset and for each continent separately. The largest amount of data was available for the temperate-continental climate (460), followed by the temperate-oceanic climate (258) and the tropics-warm humid climate (104). Only 80, 44, 21, 12 and 6 data were collected for the subtropical-summer rains, subtropical-winter rains, tropic-seas dry, boreal and cool tropics climates, respectively.

**Table 1:** Minimal, maximal, median and mean values of nitrous oxide (N_2_O) and amount of applied N (N rate) for the world and for North America, South America, Asia, Europe and Oceania.

Variable	Continent/world	min	median	mean	max	Number of data
	World	0.003	1.07	2.4	46.44	985
	Asia	0.01	0.53	1.11	15.60	124
	Europe	0.004	1.25	2.53	31.73	453
N_2_O (kg N ha^−1^ yr^−1^)	North America	0.004	0.93	2.16	26.9	306
	Oceania	0.016	1.39	2.45	15	26
	South America	0.003	1.56	4.67	46.44	76
	World	0	100	124	500	985
	Asia	0	120	139.8	423	124
	Europe	0	100	132	500	453
N rate (kg N ha^−1^)	North America	0	92	115.3	450	306
	Oceania	0	66	108.6	500	26
	South America	0	0	90.96	360	76

## Statistical Analysis

### Statistical Models

Thirteen models relating N_2_O emission to the amount of applied N were fitted to the data (Table 2). These models were characterized by (i) the presence or absence of the explanatory variable “applied N” (X), (ii) the function relating emission to applied N (an exponential or linear function), (iii) fixed or random background emission (i.e., emission for X = 0), and (iv) fixed or random N effect.

**Table 2:** Characteristics of the 13 statistical models for N_2_O emission.

Model name	Linear	Amount ofN applied	Intercept	Effect of the amount of N applied	AIC	% AIC	BIC	% BIC	DIC
NL-N-FF	No	Yes	Fixed	Fixed	5513.1	23.0	5527.8	22.6	–
NL-0-R	No	No	Random	–	5091.9	13.6	5106.5	13.3	–
NL-N-RF	No	Yes	Random	Fixed	4553.9	1.6	4573.5	1.5	–
NL-N-FR	No	Yes	Fixed	Random	4598.9	2.6	4618.5	2.5	–
NL-N-RR	No	Yes	Random	Random	4482.7	0	4507.1	0	–
NL-N-RR-B	No	Yes	Random	Random	–	–	–	–	4196.71
L-0-F	Yes	No	Fixed	–	5653.9	20.5	5663.7	20.1	–
L-N-FF	Yes	Yes	Fixed	Fixed	5512.1	17.4	5526.8	17.2	–
L-0-R	Yes	No	Random	–	5268.5	12.3	5283.2	12.0	–
L-N-RF	Yes	Yes	Random	Fixed	5117.4	9.0	5136.9	8.9	–
L-N-FR	Yes	Yes	Fixed	Random	4698.0	0.1	4717.5	0	–
L-N-RR	Yes	Yes	Random	Random	4693.2	0	4717.6	0.002	–
L-N-RR-B	Yes	Yes	Random	Random	–	–	–	–	4421.63

Models were characterized by their response function (linear or exponential), the use of the explanatory variable ‘amount of applied N’, the use of random effects for the intercept and/or the effect of the amount of N applied, values of the Akaïke and Schwartz criteria (AIC and BIC), and of the deviance information criterion (DIC) for Bayesian models. % AIC and % BIC indicate the percentage increase in AIC and BIC with respect to the best linear and nonlinear models.

The first 11 models (with L, NL, N, 0, F, and R standing for linear, nonlinear, nitrogen effect, no nitrogen effect, fixed parameter and random parameter, respectively) can be expressed as:

Model NL-N-FF: (1) 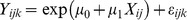


with 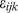
∼
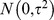


Model NL-0-R: (2) 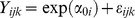


with 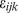
∼
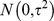
and 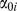
∼
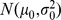


Model NL-N-RF: (3) 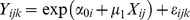


with 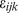
∼
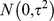
and 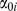
∼
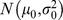


Model NL-N-FR: (4) 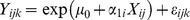


with 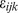
∼
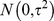
and
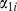
∼
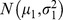


Model NL-N-RR: (5) 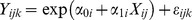


with 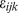
∼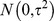, 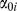
∼
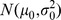
and 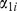
∼
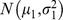


Model L-0-F: (6) 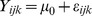


with 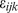
∼
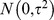


Model L-N-FF: (7) 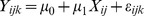


with 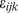
∼
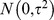


Model L-0-R: (8) 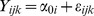


with 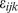
∼
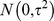
and 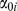
∼
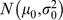


Model L-N-RF: (9) 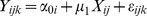


with 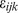
∼
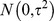
and 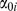
∼
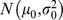


Model L-N-FR: (10) 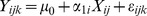


with 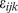
∼
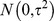
and 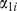
∼
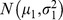


Model L-N-RR: (11) 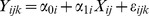


with 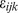
∼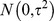, 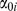
∼
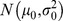
and 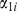
∼
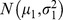
where *Y_i_*_jk_ is the N_2_O emission (kg N ha^−1^ yr^−1^) measured in the *i* ^th^ published experiment (*i* = 1 … 203), the *j* ^th^ applied N dose (*j* = 1 … *N_i_*), and the *k* ^th^ replicate (*k* = 1 … *K_ij_*), *X_ij_* is the *j* ^th^ applied N dose (kg N ha^−1^) in the *i* ^th^ published experiment, μ_0_ is the mean background emission, α_0i_ is the published experiment-specific background emission (random), μ_1_ is the mean applied N effect, α_1i_ is the published experiment-specific applied N effect (random), and ε_ijk_ is the residual error term. The random terms α_0i_, α_1i_ and ε_ijk_ were assumed to be independent and normally distributed. Models including correlated α_0i_ and α_1i_ were also fitted to the data but, as their outputs were very similar to the outputs of the models with independent random parameters, they were not considered further. Note that, in nonlinear models (1–5), the N_2_O response does not follow a normal distribution, even if its parameters α_0i_ and α_1i_ do, due to the use of an exponential function to relate emissions to model parameters.

In the linear models (6–11), the parameter μ_1_ corresponds to the emission factor EF used by the IPCC. In the nonlinear models based on an exponential function (1–5), N_2_O emission per unit of applied N is not constant; instead, it increases as a function of X if μ_1_ is positive. In the models including one or two random parameters (2–5 and 8–11), the response of N_2_O to the amount of applied N is assumed to follow the same function (linear or exponential) in all experiments, but the parameters of these models (background emission α_0i_, effect of applied N α_1i_, or both) were assumed to vary between experiments. Distributions of α_0i_ and α_1i_ describe the between-experiment variability of background emission and N fertilizer effect. An intercept was included in all statistical models to account for background anthropogenic N_2_O emission [18]. The values of the μ_0_, μ_1_, σ_0_, σ_1_, and τ parameters of models 1–11 were estimated by an approximate maximum likelihood method, with the nlme R statistical package [29].

Two additional models, NL-N-RR-B and L-N-RR-B, were defined. These models were based on the equations of models NL-N-RR and L-N-RR, respectively, but their parameters were estimated by a Bayesian method implemented with a Markov chain Monte Carlo algorithm (MCMC). Normal and independent prior probability distributions were defined for μ_0_ and μ_1;_ μ_0_, μ_1_∼N(0,1000). Uniform and independent prior probability distributions were defined for τ, σ_0_, σ_1_; τ, σ_0_, σ_1_∼U(0,100). Under these assumptions, μ_0_ and μ_1_ had a prior mean of zero and a prior standard deviation of 32, which is quite large given the measured values, which ranged from 0.003 to 46.44 in our dataset. These distributions represent a broad *a priori* distribution with respect to the data obtained. For example, the 95% credibility interval derived from the prior distributions ranged from −6272.3 to 6331.8 N_2_O kg N ha^−1^ yr^−1^ for X = 100 kg N ha^−1^. Posterior distributions of the parameters of models NL-N-RR-B and L-N-RR-B were calculated with WinBUGS software [30], with three chains of 100,000 MCMC iterations. Convergence was checked with the Gelman-Rubin method [31].

### Model Assessment and Uncertainty Analysis

The Akaike information criterion (AIC) and the Schwartz criterion (BIC) [32], [33] were calculated for the first 11 models, and the deviance information criterion (DIC) [34] was calculated for the two Bayesian models. Lower values of AIC, BIC or DIC are considered to indicate better models. Note that the weighting of the experiments according to their lengths did not reduce AIC, BIC or DIC.

We calculated the 95% confidence intervals for each model by a bootstrap method [35], [36]; data were sampled, with replacement, 500 times, and each model was fitted to each of the generated samples. For the two Bayesian models, 95% credibility intervals for the predicted N_2_O emissions were calculated from the parameter values generated by the MCMC algorithm.

The predictions generated by the three best non-Bayesian linear models, the three best non-Bayesian exponential models (selected with AIC and BIC criteria) and the two Bayesian models were compared with the N_2_O emissions calculated by the IPCC-Tier1 method: Y = EF*X, where EF is taken as 0.01 [14]. The range of uncertainty on predicted N_2_O emissions for the IPCC method was calculated from the minimum and maximum values of EF (0.003 and 0.03, respectively) reported by the IPCC [14]. The emissions due to applied N calculated with the IPCC method were compared with the predictions of the eight selected models minus the values predicted at *X* = 0.

This uncertainty range was then compared with each of the confidence intervals for the eight selected models. We also compared the lower limit of the IPCC uncertainty range with the lowest of the eight 2.5 percentiles calculated for the eight selected models, and the upper limit of the IPCC uncertainty range with the highest of the eight 97.5 percentiles of the eight selected models. The most extreme 2.5 and 97.5 percentiles obtained with the eight selected models can be interpreted as best-case and worst-case emission scenarios, respectively. They correspond to the lowest and highest limits of the confidence intervals calculated for the eight models.

The code used for statistical analysis is available, on request, from the corresponding author.

## Results

### Parameter Values

The estimated value of parameter μ_0_ (mean background emission) ranged from −0.21 to 0.88 for nonlinear models and from 0.69 to 2.78 for linear models (Table 3). The between-model variability of the estimated values of μ_1_ (mean applied N effect) was small: estimated values ranged from 0.0033 to 0.0050 for nonlinear models and from 0.0113 to 0.0138 for linear models. For both linear and nonlinear models, the estimated values of μ_1_ were lower when the effect of applied N was considered a random effect (Table 3). For example, the estimated value of μ_1_ was 0.005 for NL-N-RF, but only 0.0037 for NL-N-RR.

μ_0_ was less accurately estimated than μ_1_; the coefficient of variation (standard deviation/estimated value) was lower for μ_1_ than for μ_0_. For a given type of function (linear or exponential) estimates of σ_0_ and σ_1_ (between-experiment standard deviation of background emission and applied N effects, respectively) were similar between models. The estimated values of τ (standard deviation of model residuals) were lower for models with random parameters and for those containing the explanatory variable X.

**Table 3:** Estimated values of the parameters of the 13 models.

Model name	μ_0_	σ_0_	μ_1_	σ_1_	τ
NL-N-FF	0.25 (0.096)	–	0.0042 (0.0003)	–	3.96
NL-0-R	0.87 (0.077)	0.84	–	–	2.84
NL-N-RF	−0.068 (0.092)	0.83	0.0050 (0.0003)	–	2.09
NL-N-FR	0.31 (0.068)	–	0.0033 (0.0005)	0.0043	2.13
NL-N-RR	0.19 (0.09)	0.72	0.0037 (0.0004)	0.0025	1.94
NL-N-RR-B	−0.21 (0.13)	0.92	0.0038 (0.0005)	0.0032	1.91
L-0-F	2.40 (0.14)	–	–	–	4.26
L-N-FF	0.69 (0.19)	–	0.0138 (0.0011)	–	3.96
L-0-R	2.78 (0.27)	3.44	–	–	2.91
L-N-RF	0.99 (0.28)	3.16	0.0130 (0.0010)	–	2.67
L-N-FR	1.09 (0.11)	–	0.0113 (0.0017)	0.0195	2.12
L-N-RR	1.04 (0.13)	0.70	0.0117 (0.0017)	0.0187	2.08
L-N-RR-B	1.04 (0.14)	0.76	0.0117 (0.0017)	0.0189	2.08

The standard deviations of the estimators of μ_0_ and μ_1_ are indicated in brackets.

### Model Selection

The lowest AIC and BIC values were obtained for the nonlinear model including two random effects (NL-N-RR) (Table 2). Thus, models based on an exponential function outperformed models based on a linear function. This result was confirmed by the DIC values obtained for the two Bayesian models: DIC was lower with the exponential function.

AIC and BIC values were much higher in models in which applied N (X) was not included as an explanatory variable. The AIC and BIC values of the NL-0-R model were 5091.9 and 5106.5, respectively, whereas the AIC and BIC values of the NL-N-RF model were 4553.9 and 4573.5, respectively (Table 2).

Models including one or two random effects outperformed those including only fixed effects. The best linear model was L-N-RR on the basis of AIC, and L-N-FR, on the basis of BIC. The NL-N-FF (no random effect) model had an AIC of 5513.1 and a BIC of 5527.8, whereas both these values were much lower (AIC = 4482.7 and BIC = 4507.1) for the NL-N-RR (two random parameters) model. Models including one or two random effects had similar AIC and BIC values; the use of one random effect rather than two did not increase AIC and BIC by more than 2.6% and 9% for the nonlinear and linear models, respectively (Table 2).

The AIC and BIC values of models (1), (2), (6), (7) and (8) were more than 10% higher than those for the best nonlinear and linear models, and were therefore not considered further. We therefore considered only models (3), (4), (5), (9), (10) and (11) and the two Bayesian models in our subsequent estimations of N_2_O emission.

### Estimation of N_2_O Emissions with the Selected Models

Figure 1 shows N_2_O emissions estimated with the eight selected models. The rate of increase of N_2_O emissions with the amount of N applied was greater with the NL-N-RF model (Fig. 1A) than with the other two nonlinear models (NL-N-FR and NL-N-RR). The predicted increase in the amount of N_2_O emitted per unit increase in the amount of applied N was thus lower when the effect of applied N was defined as a random effect. Similar results were obtained for linear models, for which the highest rate of increase in N_2_O emissions with the amount of N applied was obtained for the L-N-RF model, which had a fixed slope (Fig. 1B). These results are consistent with the estimated parameter values reported in Table 3.

The emissions predicted by the Bayesian model NL-N-RR-B were the lowest for all values of applied N (Fig. 1A), due to the low estimated value of the intercept for this model (Table 3). The amounts of emission predicted by the L-N-RR model and its Bayesian counterpart (L-N-RR-B) were very similar and were essentially undistinguishable.

Figure 2 shows the fitted response curves obtained with the best linear and nonlinear models, NL-N-RR and L-N-RR, for four experiments. Considering experiment-specific responses, the nonlinear model better fitted the emissions measured at high N doses in experiments 1, 2, and 4, and the emissions measured at low N doses in experiments 3 and 4. Between-experiment variability was high for N_2_O emissions (Figure 2) and could be accounted for by the experiment-effects included in the mixed-effect models. The residual standard error was lower with NL-N-RR than with L-N-RR (see values of τ in Table 3).

### Comparison with the Emissions Estimated with the IPCC-Tier 1 Method

**Figure 3:**
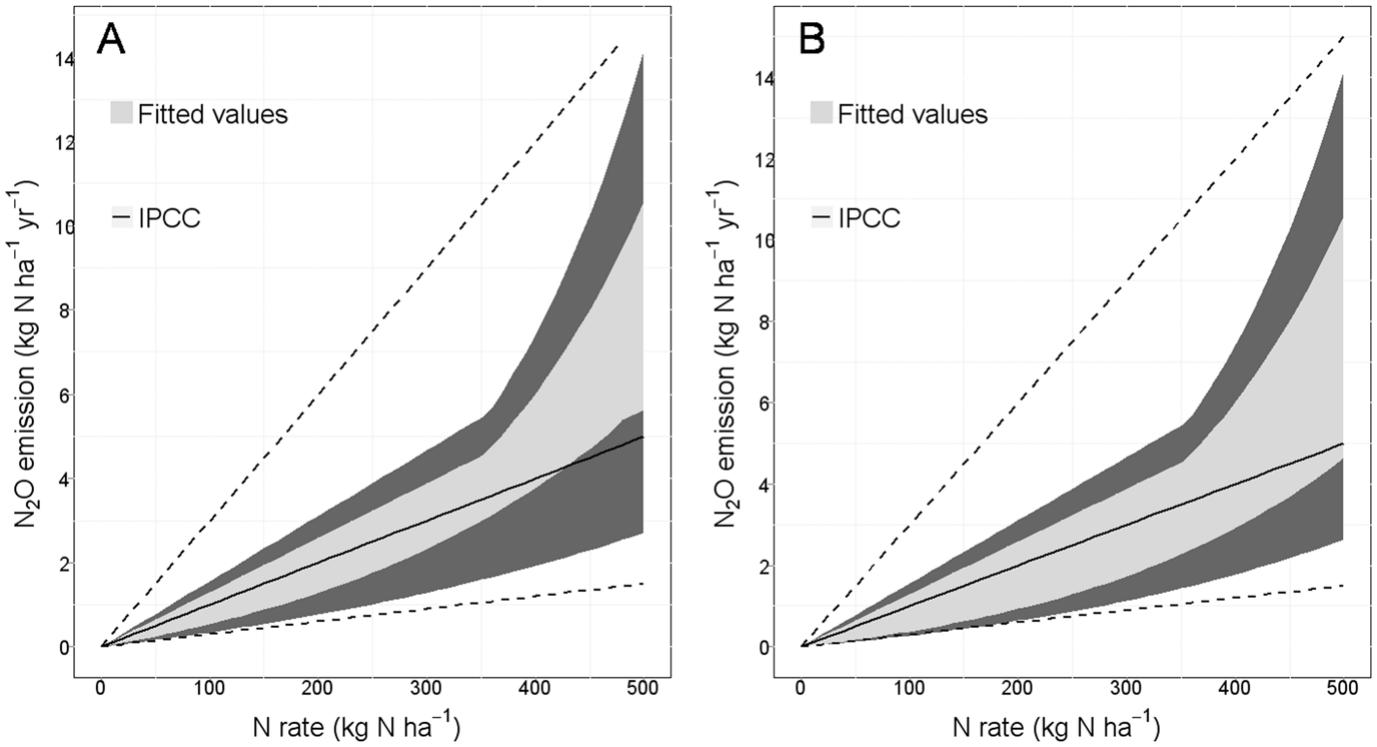
Predicted N_2_O emissions and uncertainty ranges for our eight selected models and the IPCC-Tier 1 method. The light gray area represents the uncertainty in the model equations and includes the mean values predicted by six models (A) or by eight models (6 non-Bayesian models +2 Bayesian models) (B). The dark gray area represents the uncertainty in model equations and parameter values. The upper and lower limits of the dark gray area indicate the worst-case and best-case scenarios, respectively, defined from six models (A) or from eight models (6 non-Bayesian models +2 Bayesian models) (B). The solid black line and the dotted lines indicate the N_2_O emissions predicted with an EF of 1% and the uncertainty range of the IPCC-Tier1 method, respectively.

We determined the ranges of N_2_O emissions (Fig. 3) covered by the eight models considered in Figure 1, either taking into account the uncertainty on the estimated parameter values (dark gray area) or not taking this uncertainty into account (light gray area). The final values predicted by the models were calculated by subtracting the predicted value at X = 0 (background emission) from the value actually predicted for a given amount of applied N. This graphical presentation made it possible to compare our models with the N_2_O emissions predicted with the IPCC-Tier 1 method. The estimates of N_2_O emission obtained with an emission factor of 1% (as used by the IPCC) were within the range of values covered by the eight selected models (Fig. 3B), but the range of uncertainty for emissions estimated with the IPCC-Tier 1 method was larger than that for the eight selected models. The upper limit of the uncertainty range for the IPCC method was much higher than that defined by the highest value of the eight 97.5 percentiles of the eight selected models, particularly for N applications below 300 kg ha^−1^, as generally practiced in farmers’ fields (Fig. 3). The lower limits of the uncertainty ranges for the IPCC method and for our models were more similar.

**Figure 4:**
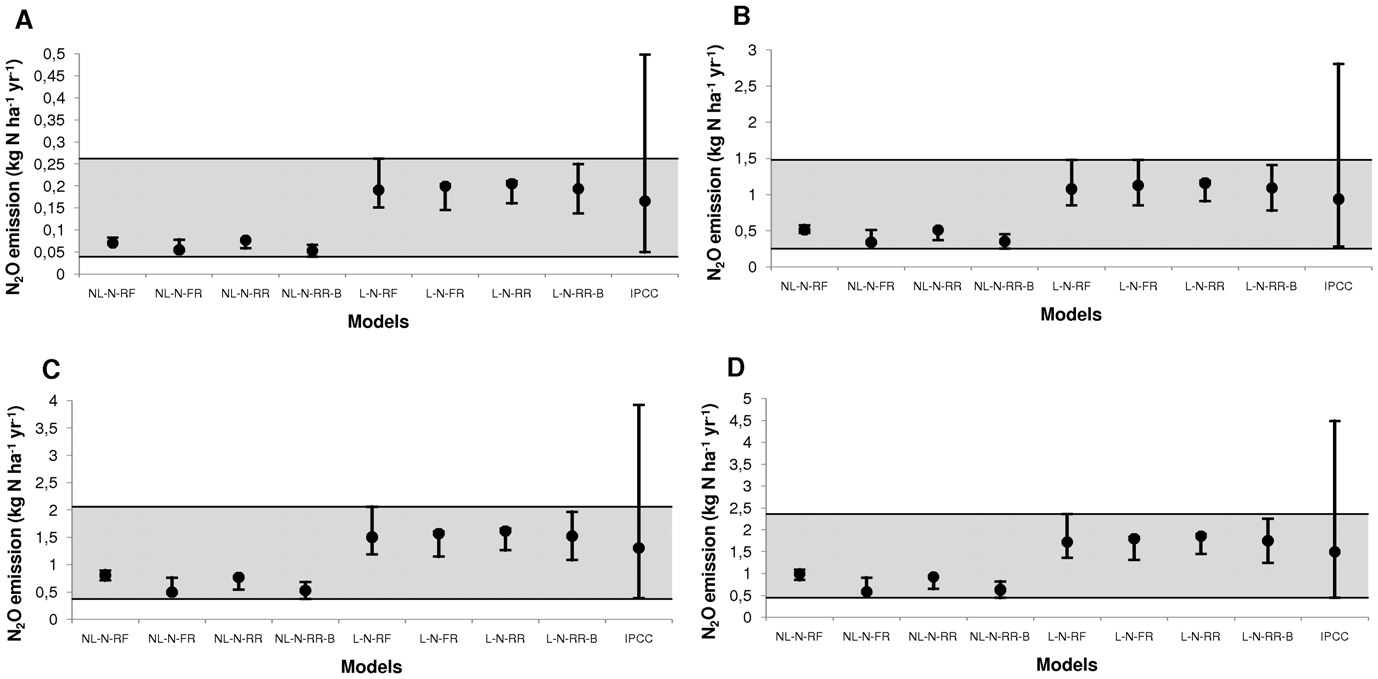
Predicted N_2_O emissions due to N fertilization and 95% confidence intervals (CI) for each model, and predicted values and uncertainty ranges for the IPCC-Tier 1 method. The light gray area corresponds to the values covered by the 95% CI of our eight models. The amounts of N applied were A) 16.62 kg N ha^−1^, B) 93.6 kg N ha^−1^, C) 130.74 kg N ha^−1^, D) 149.58 kg N ha^−1^ (average amounts of applied N for western, eastern and southern Africa, worldwide, Europe, and eastern Asia respectively). N_2_O emissions were estimated by subtracting the value corresponding to the application of no N from the value for each amount of N applied.

This result was confirmed (Fig. 4) by comparing the estimates of N_2_O emissions due to applied N obtained with the eight models with those obtained by the IPCC-Tier 1 method for four different amounts of applied N. These amounts of applied N correspond to the average amounts applied in western, eastern and southern Africa, worldwide, Europe and eastern Asia [10]. The purpose of Figure 4 was to compare model predictions for contrasted applied N doses, not to calculate average emissions at the continental scale. The uncertainty ranges obtained with the IPCC method were indeed larger than those defined by the highest of the 97.5 percentiles and the lowest of the 2.5 percentiles for the eight selected models (Fig. 4). The upper limits of the IPCC uncertainty ranges were systematically higher than the highest 97.5 percentile obtained with our models. We also found that the emissions predicted by the IPCC method were very similar to those obtained with the linear models, but systematically higher than the emissions predicted by the nonlinear models (Fig. 3).

## Discussion

Our analysis was carried out with the dataset of Stehfest and Bouwman [10] because this dataset includes a large number of data of N_2_O emissions in agricultural soils. These data were collected under various conditions characterized by different measurement methods (e.g. from short to long periods of measurements), different soils, climates, and crops. The variability of these conditions and their effects on N_2_O emission were taken into account in our analysis using random parameter models. In these models, the N_2_O emission was related to applied N using linear or nonlinear functions including two random parameters (α_0i_ and α_1i_). The probability distribution of these parameters describes the between-study variability of the parameter values due to i) the heterogeneity of the experimental protocols (i.e. length of the experiment and measurement frequency) and ii) the variability of soil, climate, and crop characteristics. This approach accounts for both the heterogeneity of the measurement protocols and the variability of the environments.

Exponential models outperformed linear models for the three statistical criteria considered: AIC, BIC and DIC. However, the differences were small. The AIC of the best linear model was only 4.7% higher than the AIC of the best exponential model. The use of an exponential function rather than a linear model has an important practical consequence: EF is not constant and increases as a function of applied N.

Our results indicate that EF is lower than the estimated value used by the IPCC-Tier 1 (i.e., 1% of applied N) if the amount of N applied is below 160 kg N ha^−1^ for the NL-N-RF model, 240 kg N ha^−1^ for the NL-N-FR model and 220 kg N ha^−1^ for the NL-N-RR model. According to Spiertz [27], farmers may apply amounts of nitrogen fertilizer below these thresholds in ecological low-input cropping systems and in some technological high-input systems. Consequently, the use of an exponential model rather than a linear model is likely to decrease estimates of N_2_O emissions in many cases. According to Hoben et al. [23], the current IPCC-Tier 1 method could lead to an underestimation of N_2_O emission if the true response is exponential. Our results suggest that this is the case only for the application of large amounts of N fertilizer.

McSwiney and Robertson [22] suggested that the use of a nonlinear model instead of a linear model leads to a greater estimated reduction in N_2_O emission for a moderate reduction in the amount of applied N, with little or no yield penalty. Our results do not entirely support this statement, because little difference was observed between the two types of models for doses of up to about 200 kg N ha^−1^ (Fig. 1). For example, if the amount of applied N is decreased from 150 kg N to 120 kg N (minus 20%), the resulting reduction of N_2_O calculated with the NL-N-RR model is 0.22 kg N ha^−1^ yr^−1^, slightly less than that calculated with the current IPCC emission factor (0.01*30 = 0.3 kg N ha^−1^ yr^−1^). With the same model, the reduction induced by a decrease from 350 kg N ha^−1^ to 280 kg N ha^−1^ (minus 20%) is much larger, reaching 1 kg N ha^−1^ yr^−1^; this value is higher than the reduction calculated with the IPCC emission factor (0.01*70 = 0.7 kg N ha^−1^ yr^−1^). The estimated reduction of N_2_O emission induced by a decrease in the amount of applied N is greater with the nonlinear model than with the linear model only for high N doses.

According to the AIC and BIC values obtained, models including one or two random effects outperformed models including fixed effects only. Mixed-effect models are commonly used in meta-analysis studies [37] and are recommended for the analysis of repeated measurements on the same individuals [38]. In the dataset of Stehfest and Bouwman [10], N_2_O emissions were measured for several amounts of N applied in the same published-experiment. It was therefore appropriate to estimate N_2_O emissions with mixed-effect models including one or two random effects in our study (Fig. 2). Models including one or two random effects performed similarly (less than 10% difference in AIC and BIC values), but the estimated effect of the amount of N applied on the amount of N_2_O emitted tended to be lower when the amount of N applied was considered as a random effect.

Several models had very similar performances. We therefore used an ensemble approach based on eight models for the estimation of N_2_O emissions and the definition of uncertainty ranges. The confidence intervals obtained with the models were used to define lower and upper limits, corresponding to the best-case and worst-case scenarios, respectively. These confidence intervals represent the uncertainty in average N_2_O emissions over all experiments, but they do not describe the between-experiment variability of N_2_O emission. The range of uncertainty defined here is relevant for the Tier 1 method and useful for explorations of the consequences of N applications for average N_2_O emissions, taking into account the uncertainty due to model equations and parameter estimations. The lower limit of our uncertainty range is close to that defined by the IPCC-Tier 1, although our lower limit is slightly higher than that of the IPCC for applications of large amounts of N. Our upper limit is much lower than the upper limit of the IPCC range, particularly for total N applications below 300 kg ha^−1^, as commonly used in agriculture. Thus, the upper limit of the IPCC range gives an estimated N_2_O emission of 9 kg N ha^−1^ yr^−1^ for a dose of 300 kg N ha^−1^, whereas the upper limit of our uncertainty range (i.e., the highest upper limit of the confidence intervals of the eight models considered) gave an estimated emission value of only 4.7 kg N ha^−1^ yr^−1^. This result is consistent with the findings of Leip et al. [12], suggesting that the uncertainty on estimates of N_2_O emissions was overestimated when derived from experimental data variances, which largely compensate at large scales.

It is useful to compare our uncertainty ranges with other ranges calculated with process-based models [39], top-down methods [40] and hierarchical Bayesian models [16].

Our uncertainty range for the average N dose applied in North America – 0.49–1.88 kg N ha^−1^ yr^−1^ – is similar to the 95% confidence interval proposed by Del Grosso et al. [39] for the United States (133–304 Gg N yr^−1^ *i.e.* 0.99–2.27 kg N ha^−1^ yr^−1^ with the cropland area of North America reported by Stehfest and Bouwman [10]).

Our uncertainty range for the average N dose applied at the world scale (93.6 kg ha^−1^ of applied N, as reported by Stehfest and Bouwman [10]) (Fig. 4B) – 0.25–1.48 kg N ha^−1^ yr^−1^ – is lower and narrower than the interval proposed by Crutzen et al. [40] (2.8–4.68 kg N ha^−1^ yr^−1^). However, it is difficult to compare these intervals, due to the use of a top-down method by Crutzen et al. Furthermore, these authors did not consider direct emission due to N fertilizer only, instead also taking into account indirect emissions from leaching and atmospheric deposition [13].

The 95% confidence interval calculated by Berdanier and Conant [16] with a hierarchical Bayesian linear model is similar to our uncertainty ranges for the four regions of the world presented in Figure 4. The two intervals overlap in all four regions, but our intervals tend to have lower upper and lower limits. For example, Berdanier and Conant [16] reported an interval of 0.05–0.46 for Africa, for a N fertilizer dose of 16.62 kg N ha^−1^ [10], whereas our interval was 0.04–0.26 kg N ha^−1^ yr^−1^ for the average N fertilizer dose reported for West, East and Southern Africa by Stehfest and Bouwman [10].

When between-experiment variability was taken into account, the experiment-specific N_2_O estimated with our models covered a wider range of values. Thus, for applied N levels of 100 kg N ha^−1^ and with the NL-N-RR model, the 90% percentile for N_2_O emission was 1.79 kg N ha^−1^ yr^−1^, the 95% percentile was 2.52 kg N ha^−1^ yr^−1^ and the 99% percentile was 5.03 kg N ha^−1^ yr^−1^, all these values being higher than the 1 kg N ha^−1^ yr^−1^ of the IPCC-Tier 1 method. Thus, N_2_O emission has 1% chance to exceed 5 kg N ha^−1^ yr^−1^ for an N fertilizer dose of 100 kg ha^−1^.

The nonlinear models presented in this paper should be used with caution for estimating average N_2_O emissions at the country and continental scales. The average output value of a nonlinear model is not strictly equal to the output value obtained with the average input value. In order to calculate the average N_2_O emission in a given country with a nonlinear model, the best approach is i) to determine the distribution of applied N fertilizer doses in this country, ii) to run the model for all doses, and iii) to take the average of all the model outputs. However, this approach requires the knowledge of the distribution of N fertilizer dose.

We focused on the Tier 1 approach of the IPCC, but the proposed exponential models could be extended to take several other environmental variables, such as climatic characteristics, soil types and fertilizer type, into account. This possibility has already been explored by Lesschen et al. [13], who took several variables into account (type of fertilizer, crop residues, atmospheric deposition, land use, soil type and precipitation) and by Leip et al. [12], who calculated the stratified emission factor as a function of soil organic carbon content, fertilizer type (mineral fertilizer or manure) and weather conditions. Such variables could be included in our models, for the estimation of region-specific N_2_O emissions, taking local characteristics into account.
